# Energetic trade-offs in migration decision-making, reproductive effort and subsequent parental care in a long-distance migratory bird

**DOI:** 10.1098/rspb.2023.2016

**Published:** 2024-02-21

**Authors:** Alexander R. Schindler, Anthony D. Fox, Christopher K. Wikle, Bart M. Ballard, Alyn J. Walsh, Seán B. A. Kelly, Lei Cao, Larry R. Griffin, Mitch D. Weegman

**Affiliations:** ^1^ Department of Biology, University of Saskatchewan, 112 Science Place, Saskatoon, Saskatchewan, S7N 5E2, Canada; ^2^ Department of Ecoscience, Aarhus University, C.F. Møllers Allé 4–8, 8000, Aarhus C, Denmark; ^3^ Department of Statistics, University of Missouri, Columbia, MO 65211, USA; ^4^ Caesar Kleberg Wildlife Research Institute, Texas A&M University-Kingsville, Kingsville, TX 78363, USA; ^5^ National Parks and Wildlife Service, Dublin, D07 N7CV, Ireland; ^6^ State Key Laboratory of Urban and Regional Ecology, Research Center for Eco-Environmental Sciences, Chinese Academy of Sciences, Beijing, 100085, People's Republic of China; ^7^ University of Chinese Academy of Sciences, Beijing, 100049, People's Republic of China; ^8^ Wildfowl & Wetlands Trust, Slimbridge, Gloucester GL2 7BT, UK; ^9^ ECO-LG Limited, Crooks House, Mabie, Dumfries, DG2 8EY, UK

**Keywords:** conservation, decision-making, full annual cycle, hierarchical modelling, migration, reproduction

## Abstract

Migratory species trade-off long-distance movement with survival and reproduction, but the spatio-temporal scales at which these decisions occur are relatively unknown. Technological and statistical advances allow fine-scale study of animal decision-making, improving our understanding of possible causes and therefore conservation management. We quantified effects of reproductive preparation during spring migration on subsequent breeding outcomes, breeding outcomes on autumn migration characteristics and autumn migration characteristics on subsequent parental survival in Greenland white-fronted geese (*Anser albifrons flavirostris*). These are long-distance migratory birds with an approximately 50% population decline from 1999 to 2022. We deployed GPS-acceleration devices on adult females to quantify up to 5 years of individual decision-making throughout the annual cycle. Weather and habitat-use affected time spent feeding and overall dynamic body acceleration (i.e. energy expenditure) during spring and autumn. Geese that expended less energy and fed longer during spring were more likely to successfully reproduce. Geese with offspring expended more energy and fed for less time during autumn, potentially representing adverse fitness consequences of breeding. These behavioural comparisons among Greenland white-fronted geese improve our understanding of fitness trade-offs underlying abundance. We provide a reproducible framework for full annual cycle modelling using location and behaviour data, applicable to similarly studied migratory animals.

## 1. Introduction

Reproduction requires substantial energy expenditure during several behavioural and physiological phases of the annual life cycle (e.g. courtship, egg-laying, incubation, parental care [1–3]). In migratory species, individuals must further balance these costs with the energy requirements of migration, creating a complex set of decision-making processes. Given spatially and temporally varying resources along migratory paths, there is substantial heterogeneity in decision-making during migration both among and within animal populations [4,5]. For example, populations of elevational migrants like elk (*Cervus canadensis*) [6] and bighorn sheep (*Ovis canadensis*) [7] exhibit considerable plasticity in elevational and geographical migration distance, and many latitudinal migrant bird species demonstrate heterogeneity in energy acquisition during migration stopovers [8,9]. While previous studies have demonstrated individual variation in reproductive success of migratory animal populations [10,11], the degree to which decisions throughout the annual cycle lead to differences in reproductive outcomes is often poorly understood. Studying migratory animals throughout their full annual cycle allows for improved understanding of their ecology, which could inform targeted conservation actions to demonstrably improve survival and reproductive success.

Recent advancements in technology to record an animal's movements, behaviour, physiology and environment, as well as statistical advancements to analyse these data have provided unprecedented opportunities for studying animal decision-making throughout the full annual cycle in field settings [12,13]. Animal-borne data loggers collect many different types of information at fine spatio-temporal scales, including GPS locations [14], acceleration [15], energy expenditure [16,17], altitude [18] and internal heart rates and temperatures [19]. These data have aided researchers in testing a variety of novel hypotheses about behavioural and physiological responses of animals to their environments. For example, recent studies have filled information gaps regarding animals' physiological responses to environmental conditions [19], predatory–prey dynamics [20], foraging niche overlap [21], habitat selection [22] and metabolic energy expenditure [23]. New knowledge in behavioural ecology is especially important for species of conservation concern because it informs conservation management actions.

The Greenland white-fronted goose (*Anser albifrons flavirostris*) is a relatively long-lived migratory species of conservation concern. Greenland white-fronted geese migrate each spring and autumn between breeding areas in west Greenland, staging areas in west and southern Iceland, and wintering areas in Great Britain and Ireland, an annual migration totalling *ca* 5000 km [24–26]. To prepare for two bouts of long-distance migration and breeding, individuals engage in intensive feeding and lower energy expenditure from late winter to the early breeding period [27–30]. However, preparation may be constrained by environmental conditions at each of these stages. For example, many bird species feed more when temperatures are low to maintain internal body conditions to survive [31,32], or expend more energy when flying through severe storms [33]. Feeding opportunities and energy expenditure may also be affected by availability and quality of food [34], as well as frequency of disturbances in feeding habitats [35,36] and agonistic interactions within and between species. Variation in environmental conditions may therefore cause heterogeneity in breeding decisions and outcomes.

The Greenland white-fronted goose population is at its lowest since the 1980s [37], having declined by approximately 50% from 1999 to 2022, and declining breeding success is the likely cause [38,39]. From 1983 to 2009, only 8% of marked individuals were observed on wintering areas with offspring at least once during their lifetimes, and only 2% were observed with offspring more than once, suggesting unusually low reproductive success among Arctic-nesting geese [40]. Furthermore, Greenland white-fronted geese maintain long-term parent–offspring associations (1–13 years), with adults likely to guide offspring on migration and teach them profitable foraging techniques [41]. This investment of parental care could increase risks to parent survival, such as through increased time spent vigilant (i.e. potentially less time spent feeding; [42,43]) or use of sub-optimal resources [44]. In many long-lived bird species, individuals lower or forgo breeding efforts in a particular breeding season when environmental conditions are poor to increase their own survival and future reproductive potential [45–47]. It is plausible that low reproductive output in Greenland white-fronted geese may in part be the result of individuals deferring breeding attempts in years of poor environmental conditions [40]; however, further study of Greenland white-fronted goose decision-making throughout the annual cycle is needed to better understand these trade-offs.

Using paired data from GPS-acceleration (ACC; i.e. dynamic movement) tracking devices deployed on Greenland white-fronted geese and remotely sensed weather and habitat data, we tested hypotheses about the fitness consequences of animal decision-making throughout the full annual cycle. We hypothesized that Greenland white-fronted geese would be more likely to defer reproductive attempts when poor environmental conditions during spring prevented adequate breeding preparation in order to avoid subsequent survival costs associated with caring for offspring. Specifically, we predicted that time spent feeding would increase with increasing number of days below freezing, decreasing precipitation, and increasing time spent in foraging habitats including grasslands, agricultural fields and peat bogs during spring and autumn migration. We predicted that energy expenditure would increase with increasing number of days below freezing, decreasing precipitation, increasing frequency of storms, increasing time spent in habitats containing anthropogenic disturbances (i.e. grasslands and agricultural fields) and decreasing time spent in more isolated habitats (i.e. peat bogs) during spring and autumn migration. We predicted geese that spent the most time feeding and expended the least amount of energy during spring migration would be most likely to reproduce successfully, geese that spent the least time feeding and expended the most energy would be most likely to defer reproduction, and geese in intermediate condition would be most likely to attempt to breed but fail. We also predicted that geese travelling with offspring during autumn migration (i.e. geese that successfully reproduced the previous breeding season) would spend less time feeding because they were more alert to protect offspring and expend more energy (from increased flights associated with avoiding disturbances or suboptimal foraging patterns), and that these altered behaviours would decrease parent survival probability.

This analysis will help us disentangle decision-making processes undertaken by individuals balancing the energy requirements of migration and breeding in a dynamic environment. Better understanding these processes for a population of conservation concern like the Greenland white-fronted goose will help us identify the drivers of poor reproductive success and inform plausible conservation actions to improve them. We provide an example for hypothesis tests about relationships among land use, climate change and animal decision-making. We anticipate that our modelling framework could be similarly applied to tracking data across a variety of taxa to improve understanding of complex linkages between animal behaviour and fitness.

## 2. Methods

### (a) Capture of geese and deployment of GPS-ACC devices

Greenland white-fronted geese were captured on four wintering sites in Ireland (Lough Iron 53°36′ N 07°28′ W, Sheskinmore 54°48′ N 08°27′ W, Lough Swilly 54°58′ N 07°38′ W, Wexford Slobs 52°21′ N 06°24′ W), three in Scotland (Loch Ken 55°00′ N 04°01′ W, Islay 55°47′ N 06°15′ W, West Freugh 54°50′ N 04°56′ W) and one staging site during autumn in Iceland (Hvanneyri 64°33′ N 21°45′ W) using standard cannon-netting techniques. Approximately half of captures occurred over sites baited with barley grains. Captured geese were sexed by cloacal examination and adult females (aged on plumage characteristics) fitted with neck collar OrniTrack-N38 GPS-ACC tracking units (approx. 38 g; www.ornitela.com). These devices collected a GPS point every 15 min, a 3 s ACC burst in three dimensions at 10 Hz (i.e. 30 ACC data points per each of the *X*, *Y* and *Z*-plane axes) every 6 min, and uploaded data daily via the Global System for Mobile communication. To maximize independence in our data, we attempted to fit devices to only one individual of a family group, as families migrate together [41]. We deployed a total of 73 GPS-ACC units between 2017 and 2022, of which 49 units had 1–5 years of sufficient data (i.e. collected over 80% of potential GPS and ACC data points; see electronic supplementary material for details) for our analyses.

### (b) Defining ecologically relevant time intervals

**Figure 1.**
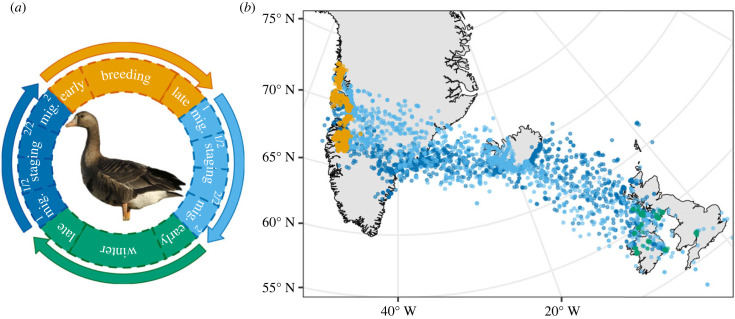
Full annual cycle of Greenland white-fronted geese. Sub-season designations used in analyses (*a*) included late winter, spring migration from wintering to Icelandic staging areas, first half of spring staging, second half of spring staging, spring migration from staging to Greenlandic breeding areas, early breeding, breeding, late breeding, autumn migration from breeding to Icelandic staging areas, first half of autumn staging, second half of autumn staging, autumn migration from staging to wintering areas, early winter and winter. Map (*b*) depicts GPS locations of all tracked geese from the study colour-coded by season (green = winter, dark blue = spring, orange = breeding, light blue = autumn).

To test hypotheses related to the fitness consequences of decision-making throughout the annual cycle, we divided the annual cycle into ecologically relevant intervals (corresponding to the spatio-temporal scales of hypothesized Greenland white-fronted goose decision-making processes). These intervals included late winter (two weeks prior to departure from wintering areas), spring flight from wintering to Icelandic staging areas, first half of spring staging, second half of spring staging, spring flight from staging to Greenlandic breeding areas, early breeding (two weeks post arrival on breeding areas), breeding, late breeding (two weeks prior to departure from breeding areas), autumn flight from breeding to staging areas, first half of autumn staging, second half of autumn staging, flight from staging to wintering areas, early wintering (two weeks post arrival on wintering areas; figure 1). We used spatial information from known breeding, staging and wintering intervals for each goose to define polygons for these regions and determined individual-specific intervals for the above periods based on timing of movements relative to these polygons (see electronic supplementary material for details).

### (c) Weather conditions

We matched GPS locations of geese in space and time with hourly surface-level temperature and barometric pressure data with a 30 m resolution from the ERA5 dataset [48] and daily cumulative precipitation data with a 0.5° latitude × 0.5° longitude resolution from the Climate Prediction Center Global Unified precipitation dataset [49] to quantify the effects of weather experienced in spring and autumn migration on time spent feeding and overall dynamic body acceleration (i.e. as a measure of energy expenditure; see §2e). During each ecologically relevant time interval in spring migration (i.e. late winter—early breeding) and autumn migration (i.e. late breeding—early winter), we calculated the proportion of days geese experienced temperatures below freezing, the average daily cumulative precipitation experienced by geese and the proportion of days during migratory flights when geese experienced severe storms (see electronic supplementary material for details).

### (d) Habitat use

We matched GPS locations of geese in space and time with 2018 Copernicus CORINE Land Cover [50] and 2018–2020 MODIS Land Cover Type Yearly Global 500 m [51] datasets to quantify the effects of habitat use during spring and autumn migration on time spent feeding and overall dynamic body acceleration. During each ecologically relevant time interval in spring and autumn migration, we calculated the proportion of time spent in grasslands (including both native grasslands and pastures), agriculture habitats (including any non-rangeland agriculture type) and peat bog habitats, as these are the primary habitats used by Greenland white-fronted geese for foraging outside of breeding areas ([25]; see electronic supplementary material for details).

### (e) Behaviour and energy expenditure

To test hypotheses relating to the effects of weather and habitat use during spring and autumn migration on decision-making, and the effects of decision-making during spring and autumn migration on breeding outcomes and survival, we used ACC data to quantify time spent feeding and overall dynamic body acceleration. We used a training dataset of known white-fronted goose behaviours linked with ACC measurements of those behaviours developed by Cunningham *et al*. [30] and VonBank *et al*. [52] and methods described in these studies to test a suite of machine learning algorithms from Nathan *et al*. [15] for classifying ACC data into behaviour. We used a random forest model (overall accuracy of greater than 95%) to classify all ACC bursts as feeding, flying, stationary or walking, summed the number of ACC fixes classified in each behaviour for each ecologically relevant time interval in spring and autumn migration, and applied the confusion matrix correction for time budgets from Resheff *et al*. [53]. We calculated overall dynamic body acceleration as a proxy for energy expenditure from ACC data [17,23] and averaged overall dynamic body acceleration values among all ACC bursts during each ecologically relevant time interval.

### (f) Arrival and departure dates

To assess the potential effects of timing of breeding area arrival on breeding outcomes and timing of breeding area departure on subsequent survival, we used GPS data to determine, across all years, the numbers of days since 31 December the preceding year each bird arrived and departed from breeding areas, respectively.

### (g) Breeding outcomes

To test hypotheses relating to the effects of decision-making during spring migration on probability of breeding outcomes, and the effects of breeding outcomes on autumn decision-making, we used GPS and ACC data during the breeding season to determine the occurrence of three potential breeding outcomes: successful breeding attempt, failed breeding attempt or breeding deferral (i.e. no breeding attempt). We used methods from Ozsanlav-Harris *et al*. [54] to calculate three metrics for identifying incubation: daily median net displacement (i.e. within-day movement), distance between successive median daily locations (i.e. among-day movement) and mean daily overall dynamic body acceleration (i.e. energy expenditure). After using a training dataset of known breeders and non-breeders to test a suite of machine-learning algorithms with these three metrics as input data, we used a random forest model (overall classification accuracy greater than 90%) to classify each day during the breeding season as ‘incubating' or not (see electronic supplementary materials for details). We defined a successful breeding attempt as 24 or more successive incubating days [55,56], a failed breeding attempt as 1–23 successive incubating days, and a breeding deferral as no incubating days.

### (h) Quantifying survival

To test hypotheses relating to the effects of decision-making during autumn migration on subsequent survival, we used GPS and ACC data during autumn migration to identify instances when a tagged bird died during autumn migration. To distinguish between instances when a bird died and instances of device failures, we calculated distances between successive mean daily locations and mean daily overall dynamic body acceleration for all individuals. We assumed that no devices were lost by tagged individuals as the neck collar design prevented device removal. We judged deaths to have occurred during autumn migration from successive days when the distance between mean daily locations was 60 m or less, and mean daily overall dynamic body acceleration was less than 0.1 (i.e. the device was transmitting but stationary for multiple days).

### (i) Statistical modelling

We combined information from all phases of the annual cycle into one hierarchical model in a Bayesian framework. This modelling framework allowed us to test hypotheses relating to the complex decision-making process that Greenland white-fronted geese undergo throughout the year in a single model, where the response variable in one phase was used to explain variation in the response variable in a subsequent phase, all while fully propagating uncertainty at each level.

### (j) Effects of spring weather and habitat use on spring behaviour

We modelled the relationships between spring weather, habitat use and energy expenditure during spring migration as a linear mixed model:

$$\log({\mathrm{ODBA}}_{i,t,s}^{\mathrm{spring}})∼N(\mu_{i,t,s}^{\mathrm{logODBA}\;\mathrm{spring}},\sigma_{1,i,t}^{2}),$$

$$\mu_{i,t,s}^{\mathrm{logODBA}\;\mathrm{spring}}=\alpha_{1,i}+\;\beta_{1,s}\times {\mathrm{freeze}}_{i,t,s}^{\mathrm{spring}}+\;\beta_{2,s}\times {\mathrm{precip}}_{i,t,s}^{\mathrm{spring}}$$

$$\begin{array}{rl} & +\beta_{3,s}\times {\mathrm{storm}}_{i,t,s}^{\mathrm{spring}}+\beta_{4,s}\times {\mathrm{grass}}_{i,t,s}^{\mathrm{spring}}+\;\beta_{5,s}\times {\mathrm{ag}}_{i,t,s}^{\mathrm{spring}}+\;\beta_{6,s}\\ & \times {\mathrm{bog}}_{i,t,s}^{\mathrm{spring}}\;\mathrm{and} \end{array}$$

$$\alpha_{1,i}∼N(0,\sigma_{2}^{2}),$$

in which $\log({\mathrm{ODBA}}_{i,t,s}^{\mathrm{spring}})$ was the log-transformed mean overall dynamic body acceleration for individual *i*, in year *t* and sub-season *s* (i.e. the corresponding ecologically relevant time interval: late winter, migration flight from winter to staging areas, first half of staging, second half of staging, migration flight from staging to breeding areas, or early breeding), *α*_1,*i*_ was an individual-specific random intercept and *β*_1−6,*s*_ were sub-season-specific effects for proportion of days below freezing (freeze), mean daily cumulative precipitation (precip), proportion of days with a severe storm (storm), proportion of time in grasslands (grass), proportion of time in agriculture (ag) and proportion of time in peat bogs (bog). We modelled the effects of spring weather and habitat use on time spent feeding as a binomial generalized linear mixed model, with the response consisting of the number of ACC bursts classified as feeding (${\mathrm{feed}}_{i,t,s}^{\mathrm{spring}}$) out of the total number of ACC bursts ($n_{i,t,s}^{\mathrm{spring}}$):

$${\mathrm{feed}}_{i,t,s}^{\mathrm{spring}}∼\mathrm{binomial}(\;p_{i,t,s}^{\mathrm{feed}\;\;\mathrm{spring}},n_{i,t,s}^{\mathrm{spring}}),$$

$$\mathrm{logit}(p_{i,t,s}^{\mathrm{feed}\;\mathrm{spring}})=\alpha_{2,i}+\;\;\beta_{7,s}\times {\mathrm{freeze}}_{i,t,s}^{\mathrm{spring}}+\;\beta_{8,s}\times {\mathrm{precip}}_{i,t,s}^{\mathrm{spring}}$$

$$\;+\beta_{9,s}\times {\mathrm{grass}}_{i,t,s}^{\mathrm{spring}}+\;\beta_{10,s}\times {\mathrm{ag}}_{i,t,s}^{\mathrm{spring}}+\;\beta_{11,s}\times {\mathrm{bog}}_{i,t,s}^{\mathrm{spring}}\;\mathrm{and}$$

$$\alpha_{2,i}∼N(0,\sigma_{3}^{2}).$$

Thus, $p_{i,t,s}^{\mathrm{feed}\;\mathrm{spring}}$ was the probability that an ACC burst was identified as feeding (i.e. proportion of time spent feeding).

### (k) Effects of spring behaviour on breeding outcomes

We hierarchically linked the mean of log-transformed spring overall dynamic body acceleration ($\mu_{i,t,s}^{\log\mathrm{ODBA}\;\mathrm{spring}}$) and logit-transformed probability of feeding in spring ($p_{i,t,s}^{\mathrm{feed}\;\mathrm{spring}}$) to the probability of breeding outcomes by:

$$B_{i,t}∼\mathrm{Categorical}(\;{\text{p}}_{i,t,k}^{B}),$$

$$\begin{array}{rl} \mathrm{logit}(p_{i,t,k}^{B})=\alpha_{3,i,k} & +\;\beta_{12,s,k}\times \mu_{i,t,s}^{\mathrm{logODBA}\;\mathrm{spring}}\\ & +\;\beta_{13,s,k}\times \mathrm{logit}(\;p_{i,t,s}^{\mathrm{feed}\;\mathrm{spring}})\\ & +\;\beta_{14,k}\times {\mathrm{arrival}}_{i,t}+\;\varepsilon_{t,k}^{\mathrm{spring}}, \end{array}$$

$$\beta_{12-13,s,k}∼N(\mu_{k}^{\beta },\sigma_{4,k}^{2}),$$

$$\begin{array}{rl} & \alpha_{3,i,k}∼N(0,\sigma_{5,k}^{2})\;\text{ and }\\ & \varepsilon_{t,k}^{\mathrm{spring}}∼N(0,\sigma_{6,k}^{2}), \end{array}$$

in which B*_i_*_,*t*_ was breeding outcome (i.e. whether a bird successfully attempted to breed, attempted to breed but failed, or did not attempt to breed), *α*_3,*i*,*k*_ was an individual-specific random intercept for breeding outcome *k*, *β*_14,*k*_ was the effect of arrival date at the breeding areas (arrival) and $\varepsilon_{t,k}^{\mathrm{spring}}$ was a year-specific random intercept. We modelled *β*_12−13,*s*,*k*_, the covariate effects of $\mu_{i,t,s}^{\mathrm{logODBA}\;\mathrm{spring}}$ and $\mathrm{logit}(\;p_{i,t,s}^{\mathrm{feed}\;\;\mathrm{spring}})$, as random sub-season deviations from a grand mean, $\mu_{k}^{\beta }$.

### (l) Effects of autumn weather, habitat use and breeding outcomes on autumn behaviour

We modelled the effects of autumn weather and habitat use on overall dynamic body acceleration and time spent feeding in the same way as in the spring portion of the model but with corresponding autumn sub-seasons (late breeding, migration flight from breeding to staging areas, first half staging, second half staging, migration flight from staging to winter areas, early winter) and with an additional fixed intercept for breeding outcome, B*_i_*_,*t*_:

$$\log({\mathrm{ODBA}}_{i,t,s}^{\mathrm{autumn}})∼N(\mu_{i,t,s}^{\log\;\mathrm{ODBA}\;\mathrm{autumn}},\sigma_{7,i,t}^{2}),$$

$$\begin{array}{rl} \mu_{i,t,s}^{\log\;\mathrm{ODBA}\;\mathrm{autumn}}=\alpha_{4,i} & +\beta_{15,s,k}\times B_{i,t}+\;\beta_{16,s}\times {\mathrm{freeze}}_{i,t,s}^{\mathrm{autumn}}\\ & +\beta_{17,s}\times {\mathrm{precip}}_{i,t,s}^{\mathrm{autumn}}+\;\beta_{18,s}\times {\mathrm{storm}}_{i,t,s}^{\mathrm{autumn}}\\ & +\beta_{19,s}\times {\mathrm{grass}}_{i,t,s}^{\mathrm{autumn}}+\beta_{20,s}\times {\mathrm{ag}}_{i,t,s}^{\mathrm{autumn}}\\ & +\beta_{21,s}\times {\mathrm{bog}}_{i,t,s}^{\mathrm{autumn}}, \end{array}$$

$$\alpha_{4,i}∼N(0,\sigma_{8}^{2}),$$

$${\mathrm{feed}}_{i,t,s}^{\mathrm{autumn}}∼\mathrm{binomial}(\;p_{i,t,s}^{\mathrm{feed}\;\mathrm{autumn}},n_{i,t,s}^{\mathrm{autumn}}),$$

$$\begin{array}{rl} \mathrm{logit}(p_{i,t,s}^{\mathrm{feed}\;\mathrm{autumn}})=\alpha_{5,i} & \;+\;\beta_{22,s,k}\times B_{i,t}+\beta_{23,s}\times {\mathrm{freeze}}_{i,t,s}^{\mathrm{autumn}}\\ & \;+\;\beta_{24,s}\times {\mathrm{precip}}_{i,t,s}^{\mathrm{autumn}}+\;\beta_{25,s}\times {\mathrm{grass}}_{i,t,s}^{\mathrm{autumn}}\\ & \;+\;\beta_{26,s}\times {\mathrm{ag}}_{i,t,s}^{\mathrm{autumn}}+\;\beta_{27,s}\times {\mathrm{bog}}_{i,t,s}^{\mathrm{autumn}}\;\text{and} \end{array}$$

$$\alpha_{5,i}∼N(0,\sigma_{9}^{2}).$$

### (m) Effects of autumn behaviour on survival

We hierarchically linked the mean of log-transformed autumn overall dynamic body acceleration ($\mu_{i,t,s}^{\mathrm{logODBA}\;\mathrm{autumn}}$) and logit-transformed probability of feeding in autumn ($p_{i,t,s}^{\mathrm{feed}\;\mathrm{autumn}}$) to autumn survival by:

$$S_{i,t}∼\mathrm{Bernoulli}(\varphi_{i,t})$$

$$\begin{array}{rl} \mathrm{logit}(\varphi_{i,t})=\alpha_{6,i} & +\;\beta_{28}\times \mu_{i,t,s}^{\log\mathrm{ODBA}\;\mathrm{autumn}}+\;\beta_{29}\times \mathrm{logit}(\;p_{i,t,s}^{\mathrm{feed}\;\mathrm{autumn}})\\ & +\;\beta_{30}\times {\mathrm{departure}}_{i,t}+\;\varepsilon_{t}^{\mathrm{autumn}}, \end{array}$$

$$\beta_{28-29,s}\;∼N(\mu^{\beta },\sigma_{10}^{2}),$$

$$\begin{array}{rl} & \alpha_{6,i}∼N(0,\sigma_{11}^{2})\;\text{and}\\ & \varepsilon_{t}^{\mathrm{autumn}}∼N(0,\sigma_{12}^{2}), \end{array}$$

in which S*_i_*_,*t*_ was 1 if the bird survived through the end of autumn migration or 0 if it died, *α*_6,*i*_ was an individual-specific random intercept, *β*_30_ was the effect of departure date from the breeding areas (departure) and $\varepsilon_{t}^{\mathrm{autumn}}$ was a year-specific random intercept. We modelled *β*_28−29,*s*_, the covariate effects of $\mu_{i,t,s}^{\log\mathrm{ODBA}\;\mathrm{autumn}}$ and $\mathrm{logit}(\;p_{i,t,s}^{\mathrm{feed}\;\mathrm{autumn}})$ as random sub-season deviations from a grand mean, *μ^β^*.

### (n) Modifications for parameterizing a breeding success model

We parameterized an alternative model by combining the breeding deferral and failed attempt categories into a ‘failed breeding' category to compare the decision-making of birds that successfully reproduced with all birds that did not. In this version, we modelled breeding success with a Bernoulli distribution (as opposed to breeding outcomes in a categorical distribution) in the spring portion of the model and included a breeding success intercept with two categories (as opposed to three) in the autumn portion of the model (see electronic supplementary material for details).

### (o) Model implementation

We fit both models to our data using Markov Chain Monte Carlo and a Gibbs sampler in JAGS [57] via the jagsUI package [58] in program R [59]. We used prior predictive checks to determine appropriate priors. We assumed $\beta_{1-11}∼N(0,1)$, $\sigma_{1}∼U(0,10)$, $\sigma_{2-3}∼U(0,1)$, $\sigma_{4-6}∼U(0,10)$, $\mu^{\beta }∼N(0,100)$, $\beta_{14}∼N(0,100)$, $\beta_{15-27}∼N(0,1)$, $\sigma_{7}∼U(0,10)$, $\sigma_{8-9}∼U(0,1)$, $\sigma_{10-12}∼U(0,10),\;\beta_{30}∼N(0,100)$. We used three chains, each with 32 000 iterations including 20 000 burn-in and thinned by 2, yielding 18 000 posterior samples for each parameter. We assessed convergence using the Gelman-Rubin statistic (R-hat < 1.1; [60]) and visual inspection of traceplots. We report the proportion of the posterior distribution above or below 0 for each *β* as evidence that *β* was positive or negative. We considered more than 70% posterior distribution above or below 0 as moderate support and more than 90% of the posterior distribution above or below 0 as strong support.

## 3. Results

### (a) Effects of weather and habitat use on behaviour during spring migration

#### (i) Weather

**Figure 2.**
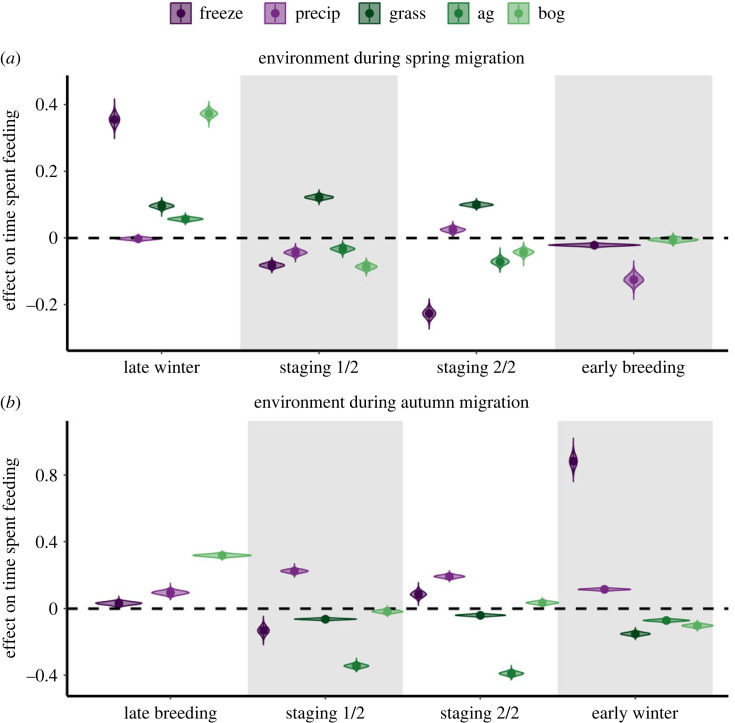
Violin plots depicting the posterior distributions, as well as the median estimates (points) and 95% credible intervals (vertical lines) for each sub-seasonal-specific effect of: (*a*) the environment on time spent feeding during spring migration and (*b*) the environment on time spent feeding during autumn migration. Purple violins represent weather covariates and green violins represent habitat use covariates; freeze, proportion of days below freezing; precip, mean daily cumulative precipitation; grass, proportion of time in grasslands; ag, proportion of time in agriculture; bog, proportion of time in peat bogs.

Days below freezing strongly affected energy expenditure (i.e. overall dynamic body acceleration) and time spent feeding during spring migration, while relationships were weaker between precipitation and energy expenditure and time spent feeding during spring migration. The number of storms negatively affected energy expenditure during spring migration flights. Geese expended more energy during late winter and the second half of spring staging (Pr(*β* > 0) = 1, 0.76, respectively) and less energy during migratory flights from wintering to staging areas, the first half of spring staging and early breeding when days below freezing were more frequent (Pr(*β* < 0) = 1, 0.82, 1; electronic supplementary material, figure S1a). Geese spent more time feeding during late winter (Pr(*β* > 0) = 1, 1) and less time feeding during both halves of spring staging and early breeding when days below freezing were more frequent (Pr(*β* < 0) = 1; figure 2*a*). Increased precipitation resulted in geese expending more energy during late winter (Pr(*β* > 0) = 0.72) and less energy during the first half of spring staging and migratory flights from staging to breeding areas (Pr(*β* < 0) = 1; electronic supplementary material, figure S1a). Geese spent more time feeding during the second half of spring staging (Pr(*β* > 0) = 1) and less time feeding during late winter, the first half of spring staging and early breeding with increasing precipitation (Pr(*β* < 0) = 0.73, 1, 1; figure 2*a*). Increased frequency of storms resulted in geese expending less energy during both spring migration flights (Pr(*β* < 0) = 0.98, 0.99; electronic supplementary material, figure S1a).

#### (ii) Habitat use

Time spent in peat bogs positively affected energy expenditure, while energy expenditure was either positively or negatively affected by time spent in grasslands, depending on sub-season. Time spent in all habitat types strongly affected time spent feeding throughout spring migration, although the directions of effects varied by sub-season. Geese expended more energy during late winter (Pr(*β* > 0) = 0.80) and less energy during both halves of spring staging with increasing time spent in grasslands (Pr(*β* < 0) = 0.95, 0.87; electronic supplementary material, figure S1a). Increased time spent in grasslands resulted in increased time feeding during late winter and both halves of spring staging (Pr(*β* > 0) = 1; figure 2*a*). Geese spent more time feeding during late winter (Pr(*β* > 0) = 1) and less time feeding during both halves of spring staging with increasing time spent in agricultural fields (Pr(*β* < 0) = 1; figure 2*a*). Geese expended more energy during late winter, both halves of spring staging and early breeding, with increasing time spent in peat bogs (Pr(*β* > 0) = 1, 0.9, 0.72, 0.91; electronic supplementary material, figure S1a). Increased time spent in peat bogs resulted in more time feeding during late winter (Pr(*β* > 0) = 1) and less time feeding during both halves of spring staging and early breeding (Pr(*β* < 0) = 1, 1, 0.84; figure 2*a*).

### (b) Effects of behaviour during spring migration on breeding outcomes

Among captured females and breeding seasons in our study period, we identified 28 successful reproductive attempts, 73 failed attempts and 7 deferrals. Geese with low energy expenditure and high time spent feeding during spring migration had the highest probability of successful breeding attempts, while low energy expenditure and low time spent feeding resulted in the highest probability of attempting to breed but failing. Geese with both high energy expenditure and low time spent feeding during spring migration had highest probability of deferring reproduction. Timing of these behaviours during spring migration did not matter; effects did not vary by sub-season.

**Figure 3.**
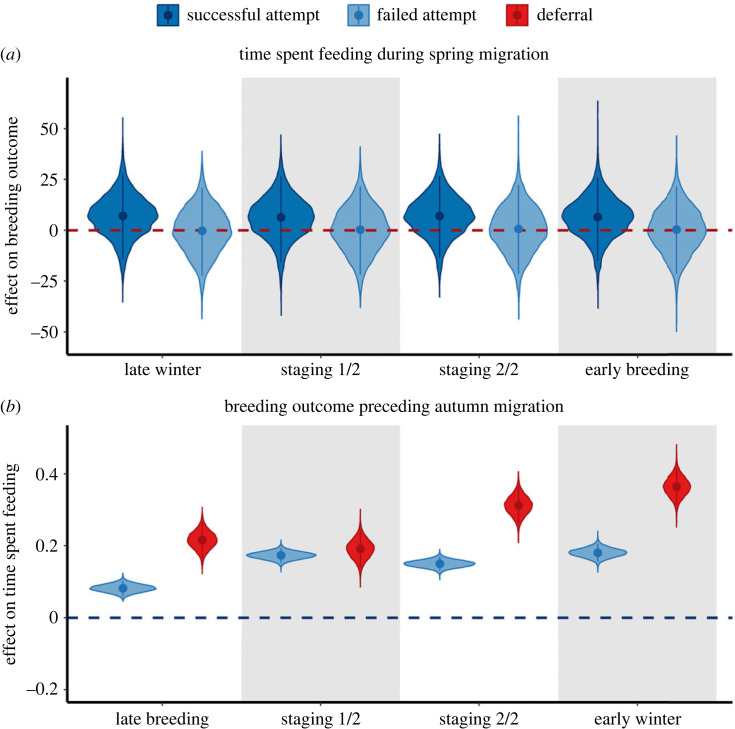
Violin plots depicting the posterior distributions, as well as the median estimates (points) and 95% credible intervals (vertical lines) for each sub-seasonal-specific effect of: (*a*) time spent feeding during spring migration on breeding outcomes (i.e. successful/failed attempts with respect to deferrals) and (*b*) breeding outcomes (i.e. failed attempts/deferrals with respect to successful attempts) on time spent feeding during autumn migration.

Geese that attempted to breed (both successful and failed attempts) expended less energy throughout migration than geese that deferred breeding (successful attempts: Pr(*β* < 0) = 0.91, failed attempts: Pr(*β* < 0) = 0.99; electronic supplementary material, figure S2a). Successfully breeding geese spent more time feeding than geese that deferred breeding (Pr(*β* > 0) = 0.78; figure 3*a*), but there was no difference in the time spent feeding among geese with failed attempts and geese that deferred breeding (Pr(*β* > 0) = 0.51; figure 3*a*). Successfully breeding birds arrived at breeding areas earlier than birds that deferred breeding (Pr(*β* < 0) = 0.73).

### (c) Effects of weather, habitat use and breeding outcomes on behaviour during autumn migration

#### (i) Weather

Days below freezing strongly affected energy expenditure and time spent feeding during autumn migration, while relationships were weaker for precipitation and energy expenditure and time spent feeding during autumn migration. Number of storms positively affected energy expenditure during autumn migration flights. Geese expended more energy during late breeding, both halves of autumn staging, migration flights from staging to wintering areas and early winter (Pr(*β* < 0) = 0.76, 1, 1, 0.86, 1) and less energy during migration flights from breeding to staging areas with increasing frequency of days below freezing (Pr(*β* > 0) = 0.71; electronic supplementary material, figure S1b). Geese spent more time feeding during late breeding, the second half of autumn staging and early winter (Pr(*β* > 0) = 1) and less time feeding during the first half of autumn staging with increasing days below freezing (Pr(*β* < 0) = 1; figure 2*b*). Increased precipitation resulted in increased energy expenditure during late breeding, the second half of autumn staging, migration flights from staging to wintering areas and early wintering (Pr(*β* > 0) = 1, 0.9, 0.87, 1) and decreased energy expenditure during the first half of autumn staging (Pr(*β* < 0.86) = 1; electronic supplementary material, figure S1b). Geese spent more time feeding during late breeding, both halves of autumn staging and early winter with increasing precipitation (Pr(*β* > 0) = 1; figure 2*b*). Increased number of storms resulted in increased energy expenditure during both autumn migration flights (Pr(*β* > 0) = 0.73, 0.99; electronic supplementary material, figure S1b).

#### (ii) Habitat use

Time spent in grasslands and agricultural fields negatively affected time spent feeding throughout autumn migration. Time spent in peat bogs affected time spent feeding and time spent in all three habitat types affected energy expenditure during autumn migration, although these relationships varied by sub-season. Geese expended less energy during both halves of autumn staging and early winter with increasing time spent in grasslands (Pr(*β* < 0) = 1, 0.97, 0.71; electronic supplementary material, figure S1b). Increased time spent in grasslands and agricultural fields resulted in less time spent feeding in both halves of staging and early winter (Pr(*β* < 0) = 1; figure 2*b*). Geese expended more energy during the second half of autumn staging (Pr(*β* > 0) = 0.98) and less energy during early winter with increasing time spent in agricultural fields (Pr(*β* < 0) = 0.84; electronic supplementary material, figure S1b). Geese expended more energy during the second half of autumn staging (Pr(*β* > 0) = 0.74) and less energy during the first half of autumn staging with increasing time spent in peat bogs (Pr(*β* < 0) = 0.78; electronic supplementary material, figure S1b). Increased time in peat bogs resulted in increased time spent feeding during late breeding and the second half of autumn staging (Pr(*β* > 0) = 1) and decreased time spent feeding during the first half of autumn staging and early winter (Pr(*β* < 0) = 0.97, 1; figure 2*b*).

#### (iii) Breeding outcomes

Geese migrating without offspring (i.e. either attempted to breed but failed or deferred the preceding breeding season) generally expended less energy during early autumn migration and spent more time feeding throughout autumn migration than geese migrating with offspring (i.e. successfully bred the preceding breeding season). Geese that deferred breeding had lower energy expenditure during migration flights from breeding to staging areas and during the first half of subsequent autumn staging (Pr(*β* < 0) = 0.93, 0.96), but higher energy expenditure during the subsequent early winter (Pr(*β* > 0) = 0.71) than successfully breeding geese (electronic supplementary material, figure S2b). Geese that attempted to breed but failed had lower overall dynamic body acceleration during late breeding, migration flights from breeding to staging areas, both halves of subsequent autumn staging and subsequent early winter (Pr(*β* < 0) = 0.80, 0.88, 0.94, 0.99, 0.87) than successfully breeding geese (electronic supplementary material, figure S2b). Geese that attempted to breed but failed on average spent 8.53% (95% credible interval (CRI) = 6.26%–10.84%), 18.93% (95% CRI = 16.43%–21.35%), 16.19% (95% CRI = 13.99%–18.48%) and 19.81% (95% CRI = 16.92%–22.80%) more time feeding during late breeding, first and second half of subsequent autumn staging, and subsequent early winter, respectively, than successfully breeding geese (Pr(*β* > 0) = 1; figure 3*b*). Geese that deferred breeding on average spent 24.07% (95% CRI = 18.2%–30.13%), 21.11% (95% CRI = 15.00%–27.43%), 36.68% (95% CRI = 29.79%–43.80%) and 43.98% (95% CRI = 36.69%–51.59%) more time feeding during late breeding, first and second half of subsequent autumn staging, and subsequent early winter, respectively, than successfully breeding geese (Pr(*β* > 0) = 1; figure 3*b*).

### (d) Effects of behaviour during autumn migration on subsequent survival

Geese that survived autumn migration expended less energy (than geese that died during autumn migration (Pr(*β* < 0) = 0.91) and these relationships did not vary by sub-season (electronic supplementary material, figure S3). Neither time spent feeding nor departure date explained substantial variation in autumn survival (Pr(*β* < 0) = 0.53, 0.60; electronic supplementary material, figure S4).

## 4. Discussion

We quantified behavioural decision-making and the relative contributions of these decisions during each phase of the annual cycle towards reproductive outcomes and subsequent survival in a long-distance migratory bird of conservation concern. We found that decisions made by female Greenland white-fronted geese during spring migration affected their probability of breeding outcomes and breeding outcomes affected decision-making during autumn migration, as parents likely chose to invest in parental care [41] at the expense of their own body condition. Furthermore, we found that environmental conditions constrained decision-making in spring and autumn migration. Greenland white-fronted geese have very low rates of breeding success [38–40] compared to similar long-lived migratory birds [10,61,62]. Some geese (approx. 6.5%) in our study may have deferred breeding attempts after experiencing poor environmental conditions that would not allow adequate preparation, thus preventing further degradation to their body condition through reproductive effort. However, the prevalence of female geese that attempted but failed to breed (approx. 67.3%) and the lack of behavioural differences during autumn migration between these and geese that deferred breeding suggest that many Greenland white-fronted geese can overcome failed breeding attempts without subsequent negative consequences. While our study sample of female geese may contain biases as we captured some geese over baited sites that were potentially dominated by higher-quality individuals, we expect a similar gradient of decision-making occurs among the entire population, where the difference between low- and high-quality individuals is likely even greater.

Many migratory birds ‘prepare' by storing energy reserves well in advance of the breeding season [63,64]. For example, snow geese (*Anser caerulescens*), pink-footed geese (*A. brachyrhynchus*) and barnacle geese (*Branta leucopsis*) all rely at least partially on energy stored prior to breeding for reproduction [65–68]. Likewise, we found that decreased energy expenditure during spring increased the probability of breeding attempts and increased time feeding during spring improved the probability of successful breeding attempts in Greenland white-fronted geese. Thus, geese that maintained a high ratio of their time spent feeding to energy expenditure prior to breeding (e.g. more time spent feeding and lower energy expenditure) had the highest probabilities of successfully breeding, while geese that were less able to attain such preparedness (e.g. low energy expenditure but less time spent feeding) often attempted but failed to breed. Additionally, some failed nests may have resulted from nest predation. While nest predation is stochastic, probability of nest predation is related to heterogeneity in individual decision-making. For example, increased frequency and duration of nest recesses increase probability of nest predation in Arctic-nesting birds [69,70]; geese that achieved optimal preparedness (i.e. that began breeding with sufficient energy stores) likely required fewer nest recesses than geese that were less able to achieve such preparedness, thus decreasing the probability of nest predation. While additional factors that we were unable to account for (e.g. age, prolonged association with parents, breeding outcomes in previous years) likely also affect breeding probabilities in individual geese, our results suggest behavioural decision-making plays a key role in determining breeding outcomes. Interestingly, the effects of decision-making (both time spent feeding and energy expenditure) on probability of breeding outcomes did not vary by time interval across spring migration. This is consistent with findings in Cunningham *et al*. [30], who examined the effects of daily decision-making on Greenland white-fronted goose breeding deferrals. We extended this framework to investigate broader intervals that we hypothesized to be of greater ecological importance, but still found similar results. These results suggest that while Greenland white-fronted geese at least partially rely on energy stored prior to breeding for successful breeding attempts, the timing of nutrient acquisition during migration does not appear to have a meaningful impact, enabling heterogeneity in migration strategies to achieve adequate body condition.

Autumn migration is a substantial cause of juvenile mortality [71] and greater white-fronted geese invest in parental care during autumn to increase juvenile survival [41], but this may have negative consequences for the condition of parents in autumn, as found in other species [40]. We found that successfully reproducing Greenland white-fronted geese increased their investment in protection of young (i.e. more time spent alert and less time spent feeding [42]) and increased energy expenditure, potentially due to increased flight times from stronger responses to disturbances or different foraging strategies (e.g. families using different foraging habitats [44]), both of which likely resulted in decreased adult body condition. We did not find evidence that decreased time feeding in successfully attempting breeders resulted in decreased survival. Family groups in goose species are socially dominant [72–74]; this may result in access to higher-quality food, allowing parents to achieve adequate body condition despite reduced feeding times or increased energy expenditure [43]. However, we are cautious in interpreting effects of behaviour on survival as a low sample size of birds that died during autumn migration (*n* = 5) may have prevented us from detecting any effects.

We found evidence that weather and habitat use affected time spent feeding and energy expenditure during both spring and autumn migration. While geese make decisions to maximize their probability of successful breeding attempts and survival, the environment can constrain these decisions. Studying how weather affects behaviour throughout the annual cycle is important for predicting how migratory animals will respond to future climate changes. For example, while we expected that geese would feed more during cold temperatures to offset energy needed to maintain internal body temperatures [31,32], we observed the opposite relationship on staging and breeding areas. Increased temperatures may be causing increased plant growth and ground thaw and decreased snow cover, leading to increased feeding opportunities during spring [30,75], and expected warming temperatures and advancement of spring may allow geese to accumulate body stores earlier [32,76]. When geese do experience cold temperatures (i.e. less available food) in these areas, they may not be able to forage efficiently and instead choose to conserve energy by remaining stationary for longer periods of time.

Studying the relative contributions of habitat use to behaviour across the annual cycle and the ultimate effects of behaviour on breeding outcomes allows us to determine the relevant time periods during which use of different habitats contributes to improved breeding success. Most habitat management for Greenland white-fronted geese occurs on wintering, and to a lesser extent, staging areas. We found that behaviour during all time periods during spring migration affected probability of successful breeding outcomes; thus, management that improves availability and quality of habitats during any of these time periods will likely lead to increased time feeding, therefore improving the likelihood of successful breeding attempts. Habitat management on wintering areas to increase availability of cereal stubble to geese and protect natural peat bog habitats, and on both staging and wintering areas to promote high-quality grasslands, will help geese spend more time feeding during these important time periods, thus arriving on breeding areas with increased probabilities of successfully breeding. Likewise, management practices that decrease disturbances of geese (e.g. agreements with agricultural producers in wintering and staging areas to delay the return of livestock to pastures or their use of agricultural machinery until geese depart for breeding areas, protection from hunting-related disturbances on wintering and staging areas, etc.) will decrease energy expenditure associated with flights in response to these disturbances, further improving the likelihood of successfully breeding.

Trade-offs in long-distance movements, reproductive effort and survival are important aspects of ecology in migratory animals, yet the mechanisms of decision-making underlying these trade-offs and the spatio-temporal scales with which they operate are often unknown. Furthermore, connecting environmental conditions and decision-making processes across breeding, migratory and wintering periods can be challenging for researchers, limiting understanding of how each part of the annual cycle differentially propagates to reproductive outcomes and survival. Given increasing pressures of land use and climate change on migratory species, improving our understanding of animal decision-making is critical to improving conservation management of these at-risk species. By combining new technologies and novel statistical methods, we can achieve unprecedented insights into animal behaviour. We believe our study will provide a framework for studying full annual cycle decision-making in any migratory species where such data are available.

## Data Availability

All data and code are available on Dryad: https://doi.org/10.5061/dryad.2547d7wzn [77]. Supplementary material is available online [78].
